# Neuromuscular magnetic stimulation counteracts muscle decline in ALS patients: results of a randomized, double-blind, controlled study

**DOI:** 10.1038/s41598-019-39313-z

**Published:** 2019-02-26

**Authors:** Antonio Musarò, Gabriella Dobrowolny, Chiara Cambieri, Emanuela Onesti, Marco Ceccanti, Vittorio Frasca, Annalinda Pisano, Bruna Cerbelli, Elisa Lepore, Gabriele Ruffolo, Pierangelo Cifelli, Cristina Roseti, Carla Giordano, Maria Cristina Gori, Eleonora Palma, Maurizio Inghilleri

**Affiliations:** 1 DAHFMO-Unit of Histology and Medical Embryology, Sapienza University of Rome, Laboratory affiliated to Istituto Pasteur Italia – Fondazione Cenci Bolognetti, Rome, 00161 Italy; 2 Center for Life Nano Science at Sapienza, Istituto Italiano di Tecnologia, Rome, 00161 Italy; 3 Rare Neuromuscular Diseases Centre, Department of Human Neuroscience, Sapienza University, Rome, Italy; 4 Department of Radiological, Oncological and Pathological Sciences, Sapienza University of Rome, Rome, 00161 Italy; 5 Department of Molecular Medicine, Sapienza University of Rome, Rome, 00161 Italy; 6 Department of Physiology and Pharmacology, Sapienza University of Rome, Laboratory affiliated to Istituto Pasteur Italia-Fondazione Cenci Bolognetti, Rome, 00185 Italy; 7 Department of Physiology and Pharmacology, Sapienza Univesrity of Rome, Rome, Italy; 8 IRCCS San Raffaele Pisana, Rome, Italy

## Abstract

The aim of the study was to verify whether neuromuscular magnetic stimulation (NMMS) improves muscle function in spinal-onset amyotrophic lateral sclerosis (ALS) patients. Twenty-two ALS patients were randomized in two groups to receive, daily for two weeks, NMMS in right or left arm (referred to as real-NMMS, rNMMS), and sham NMMS (sNMMS) in the opposite arm. All the patients underwent a median nerve conduction (compound muscle action potential, CMAP) study and a clinical examination that included a handgrip strength test and an evaluation of upper limb muscle strength by means of the Medical Research Council Muscle Scale (MRC). Muscle biopsy was then performed bilaterally on the flexor carpi radialis muscle to monitor morpho-functional parameters and molecular changes. Patients and physicians who performed examinations were blinded to the side of real intervention. The primary outcome was the change in the muscle strength in upper arms. The secondary outcomes were the change from baseline in the CMAP amplitudes, in the nicotinic ACh currents, in the expression levels of a selected panel of genes involved in muscle growth and atrophy, and in histomorphometric parameters of ALS muscle fibers. The Repeated Measures (RM) ANOVA with a Greenhouse-Geisser correction (sphericity not assumed) showed a significant effect [F(3, 63) = 5.907, p < 0.01] of rNMMS on MRC scale at the flexor carpi radialis muscle, thus demonstrating that the rNMMS significantly improves muscle strength in flexor muscles in the forearm. Secondary outcomes showed that the improvement observed in rNMMS-treated muscles was associated to counteracting muscle atrophy, down-modulating the proteolysis, and increasing the efficacy of nicotinic ACh receptors (AChRs). We did not observe any significant difference in pre- and post-stimulation CMAP amplitudes, evoked by median nerve stimulation. This suggests that the improvement in muscle strength observed in the stimulated arm is unlikely related to reinnervation. The real and sham treatments were well tolerated without evident side effects. Although promising, this is a proof of concept study, without an immediate clinical translation, that requires further clinical validation.

## Introduction

Amyotrophic lateral sclerosis (ALS) is a multi-factorial and multi-systemic pathology associated with motor neuron degeneration, muscle atrophy and paralysis^1^. Although several of the pathological mechanisms have been understood^2^, ALS remains an invariably fatal disease for which no effective therapy is known. A range of genetic and environmental conditions has been associated with ALS. Interestingly, notwithstanding the highly variable etiology of ALS, both sporadic and familial forms of this disease display a remarkable similarity in terms of disease progression and clinical manifestations. A crucial biological mechanism that is seriously affected in ALS is the loss of effective connections between muscle and nerve. Mounting evidence suggests that the earliest presymptomatic functional and pathological changes occur distally in axons and at the neuromuscular junctions (NMJ)^3,4^. These changes precede, and can be independent of, the loss of cell bodies or alterations in other cell types already linked to the ALS disease process^4–7^. In keeping with these findings, we observed that the AChRs in human ALS muscles are less sensitive to ACh than denervated non-ALS muscles^8^. It has also been reported that muscle specific expression of the mutant *SOD1* gene, one of the genes associated with the familial form of ALS, induces muscle atrophy, a significant reduction in muscle strength, mitochondrial dysfunction, microgliosis^5^ and neuronal degeneration^9^, thus suggesting that retrograde signals from muscle to nerve may contribute to synapse and axon damage. This indicates that skeletal muscle could be an important target for therapeutic interventions^7,10^. Therefore, ensuring that the muscles remain strong and active might help to maintain muscle performance in the face of dwindling motor neuron input. The neuromuscular system may be artificially stimulated either by electrical stimulation (ES) or by time-varying electromagnetic fields. It has been demonstrated that transcutaneous ES enhances mass and muscle function in the elderly by inducing anabolic pathways and negatively modulating muscular catabolism^11^. Unfortunately, ES activates cutaneous nociceptors, including A-delta high-threshold mechanoreceptors, C-fiber polymodal nociceptors and A-delta myelinated heat nociceptors, which result in painful and unpleasant sensations. Furthermore, the activation of the nociceptive pathways might evoke exaggerated cutaneous withdrawal flexor reflexes, which cause discomfort and exacerbate a patient’s spasticity^12^.

Neuromuscular magnetic stimulation (NMMS) has been proposed as an alternative, non-invasive, stimulation technique. NMMS is a painless and well-tolerated procedure that does not induce high-intensity cutaneous electric fields or activate skin nociceptors^13^. However, up to now, the effects of NMMS on muscle performance in patients with ALS have not been investigated.

In the current study, we tested the hypothesis that NMMS can improve muscle function and strength in spinal-onset ALS patients. Here we examined the effects of NMMS in patients with ALS, analyzing and comparing the morpho-functional properties and related molecular markers of both treated and untreated muscles. Data were compared with records obtained from sham NMMS. The primary outcome was the evaluation of the efficacy and safety of NMMS in improving the muscular strength of the patient affected by ALS. Secondary outcomes included the effect of NMMS on the following parameters: *i*. the electrophysiological changes (study of CMAP) to define the physiological mechanisms of rNMMS; *ii*. the analysis of the nicotinic ACh-evoked currents in muscles of ALS patients; *iii*. the histomorphometric parameters of the same ALS muscles, analyzing muscle fiber type composition, muscle atrophy and wasting; *iv*. the changes in gene expression of a selected panel of genes involved in muscle growth and atrophy. Outcome measures were completed at baseline and at the end of treatment. Potential adverse events, experienced by the patients, were monitored during the period of treatment and after 3 months follow-up period.

We demonstrate that repetitive NMMS improves muscle strength and function and activates a molecular circuit that counteracts muscle atrophy and strengthens the muscle in spinal-onset ALS patients, with a good tolerability.

## Results

Twenty-two patients were enrolled in this study (19 male, 3 female). The patients’ mean age at the time of the baseline visit was 61.27 ± 13.44 years (range 37 to 81 years), while their mean disease duration was 26.82 ± 15.43 months.

**Figure 1:**
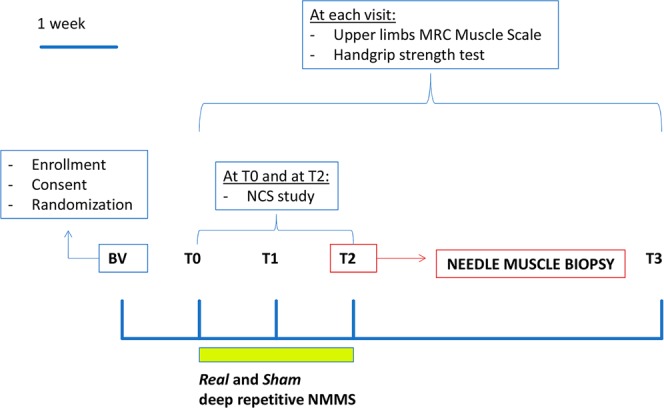
Study design. BV (baseline visit) represents the screening of ALS patients; T0 is the first recording of clinical strength and neurophysiological parameters before stimulation, T1 after one week, and T2 after two weeks of stimulation; T3 is thirty days after the end of stimulation.

**Figure 2:**
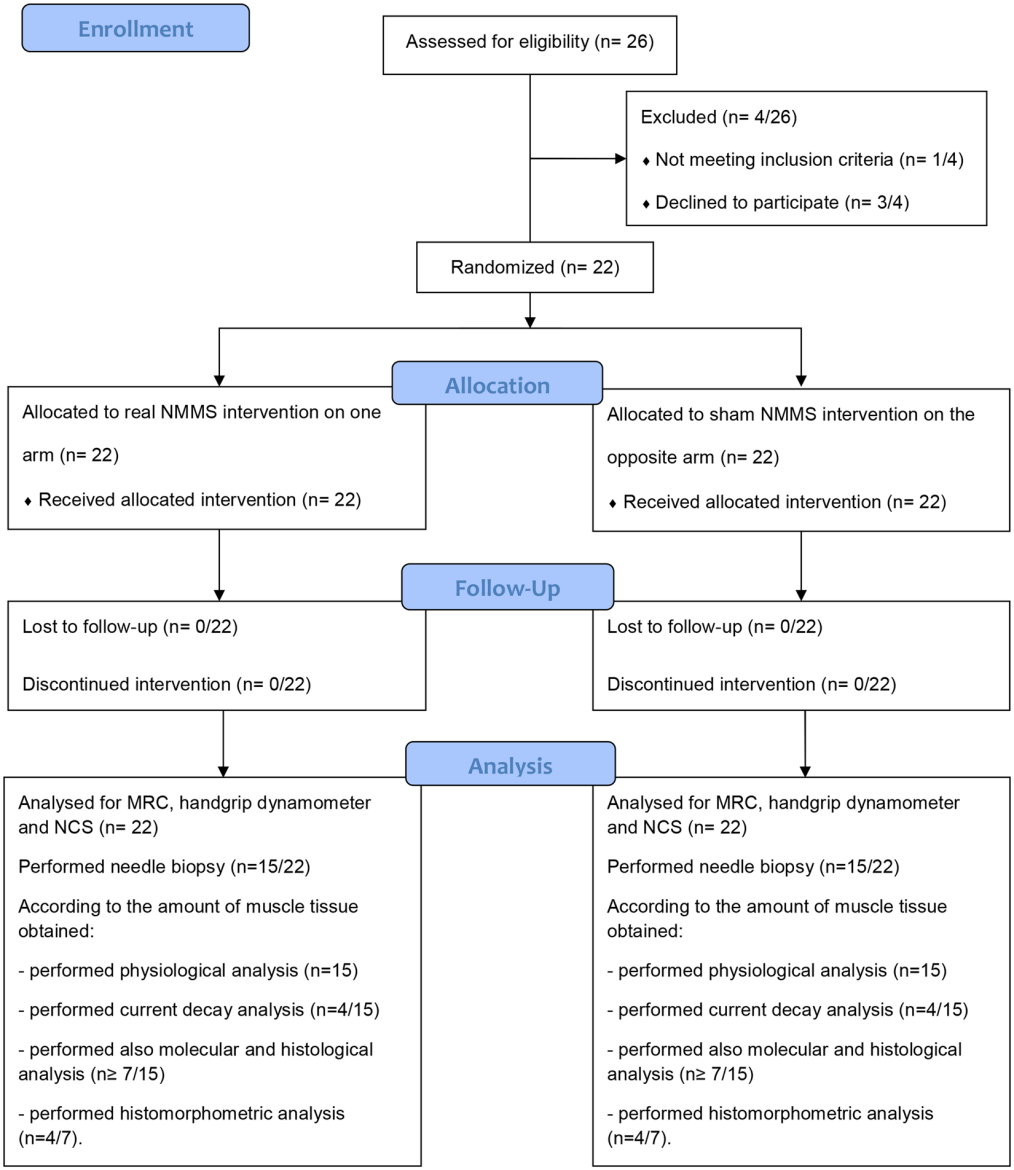
Flowchart diagram of the study.

We randomized 22 patients, who completed and well tolerated the clinical study. Out of those, only 15 patients underwent the needle-biopsy as 7 patients finally refused the procedure because of poor compliance. Unfortunately, the amount of muscle obtained with the percutaneous needle-biopsy was not always sufficient to carry out all the proposed analyses. Thus, according to the amount of muscle available 15 patients provided muscle samples for the electrophysiological study of the nicotinic AChRs, among these only 7 patients provided also samples for the molecular and the histological analysis. Additionally, 4 patients of the latter were selected for histomorphometric analysis. This selection was based on the possibility to count at least 50 fibers on a muscle cross section observed under light microscopy. The rNMMS did not evoke any significant muscle twitch and was a well-tolerated and painless stimulation procedure. The study design is represented in Fig. 1 and a flow chart showing details of patients’ allocation is represented in Fig. 2.

### NMMS improves muscle strength

**Figure 3:**
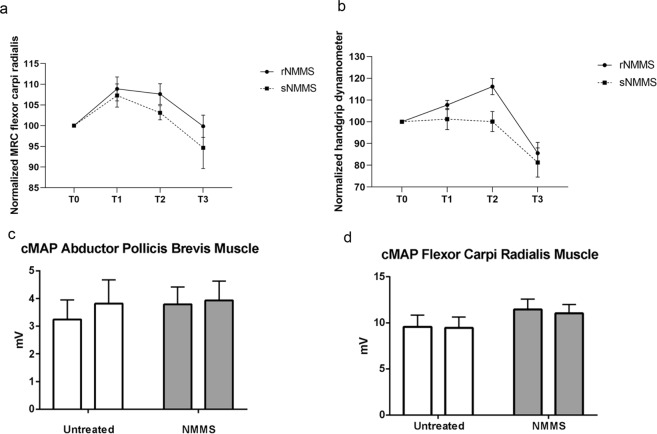
Significant improvement in muscle strength of the flexor carpi radialis muscle. (**a**) Muscle strength was tested by means of the MRC Muscle Scale and (**b**) handgrip dynamometer after NMMS. No significant improvement in muscle strength was observed in the control group. (**c**) No significant difference in cMAP amplitudes was observed before and after NMMS in the APB and (**d**) flexor carpi radialis muscles after median motor nerve stimulation at the elbow (Data represent mean ± SEM).

To determine whether rNMMS improves muscle strength, we used the MRC Muscle Scale for the upper limbs and a performed a handgrip dynamometer bilateral assessment before rNMMS (T0), at the end of rNMMS (T2) and 30 days after rNMMS ended (T3) (Fig. 3).

No significant differences in the MRC Muscle Scale at the flexor carpi radialis muscle (rNMMS: 3.95 ± 0.57 *vs* sNMMS: 3.63 ± 0.84, p = 0.31, Mann-Whitney U test) and dynamometer values (rNMMS: 22.0 ± 6.2 Kg vs sNMMS: 19.9 ± 5.8 Kg, p = 0.71, Mann-Whitney U test) were observed between treated and untreated groups at the baseline (T0).

The results of the RM ANOVA with a Greenhouse-Geisser correction (sphericity not assumed) (Fig. 3) showed that rNMMS induced a significant effect [F(1.697, 35.630) = 19.583, p < 0.01] on the muscle strength, as measured by the handgrip dynamometer; the within-subject analysis demonstrated a significant post-stimulation increase in muscle strength (measured by dynamometer) compared with the pre-stimulation (T0), both at T1 (p = 0.001), and T2 (p = 0.0001) and a significant decrease in T3 (p = 0.004). The between-subject analysis showed a significant interaction dynamometer*group of treatment at T2 (p = 0.004). The same RM ANOVA analysis, with assumption of sphericity, showed a significant effect [F(3, 63) = 5.907, p < 0.01] of rNMMS on MRC scale at the flexor carpi radialis muscle; the within-subject analysis demonstrated a significant post-stimulation increase in MRC scale, compared with the pre-stimulation strength (T0), both at T1 (p = 0.008), and T2 (p = 0.005); a significant decrease was not observed at T3 (p > 0.05). No interaction MRC*group of treatment was found at any time point.

By contrast, no significant improvement in muscle strength was observed in the other untreated muscles, tested with the dynamometer at T1 (p = 0.847) and T2 (p = 0.086); a significant reduction in the muscle strength was found at T3 (p = 0.006). In order to verify whether the improvement in muscle strength was related to a collateral sprouting of motor neurons, we performed a nerve conduction study (NCS) of the median motor nerve bilaterally; CMAPs were obtained from the APB and flexor carpi radialis muscles. No significant difference in CMAP amplitudes was observed before and after rNMMS (Fig. 3c,d).

### NMMS preserves fast muscle fibers

**Figure 4:**
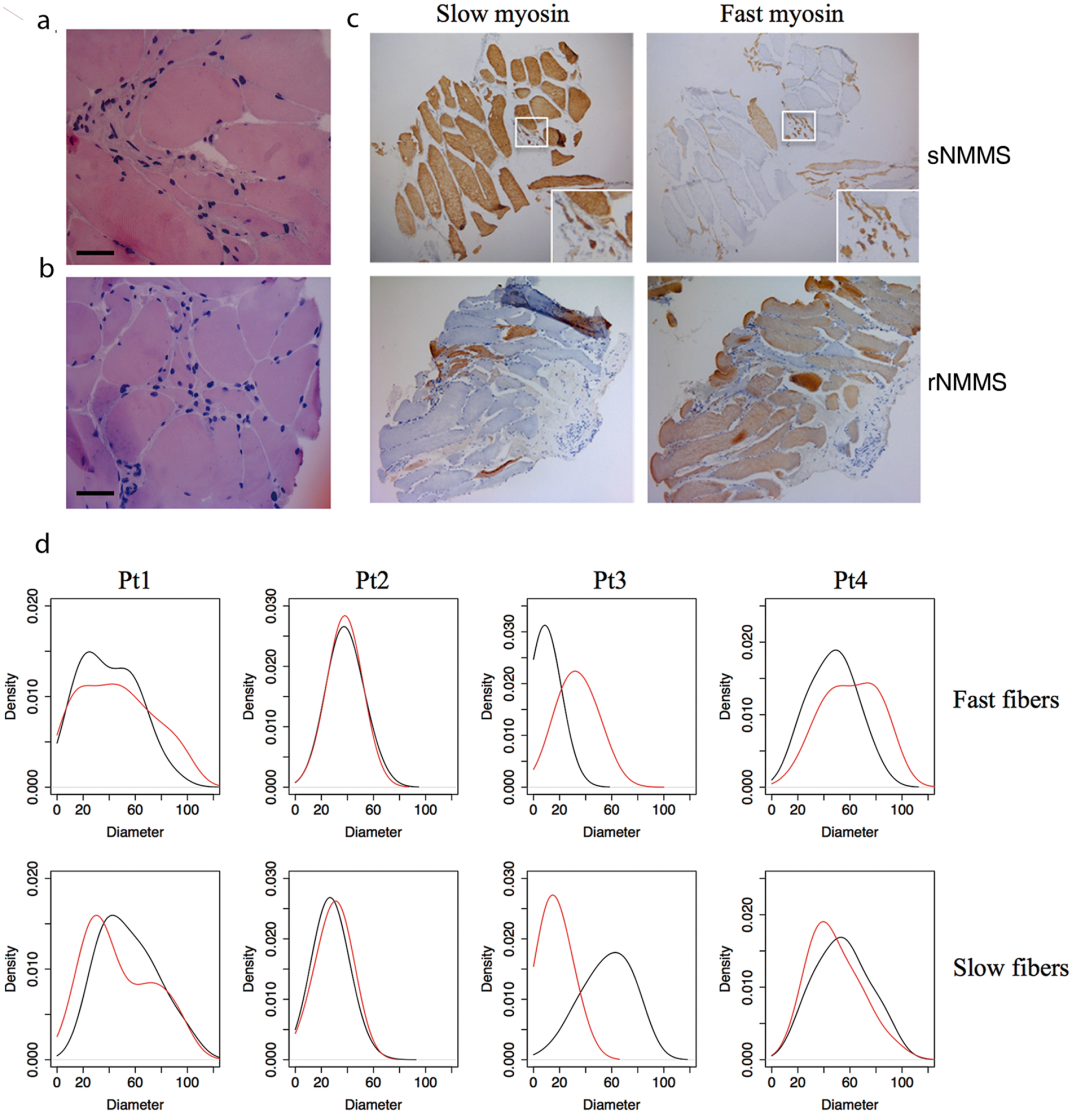
Morphologic and morphometric analysis of untreated and NMMS treated muscles. (**a**) Hematoxylin and eosin staining of muscle biopsies in sNMMS- and (**b**) rNMMS-treated muscles display grouped atrophy and scattered hypertrophic fibers (scale bar 50 µm). (**c**) Immunohistochemical staining for slow and fast myosin show that the majority of the fast fibers are atrophic in the representative sNMMS muscle (upper panel). The contralateral rNMMS-treated muscle (lower panel) is characterized by a prevalence of fast fibers. (**d**) Density estimation of the diameter for fast and slow twitch fibers across 4 patients, showing a significant shift in the peak fast fiber diameter towards higher values in rNMMS (red line) if compared with sNMMS (black line) (p < 0.001).

To verify whether the improvement in muscle strength induced by rNMMS parallels the changes in the degree of muscle atrophy and/or fiber type composition, we performed histological analyses (n = 7 patients) and morphometric measurements (n = 4 patients) on needle biopsies obtained at the end of the NMMS sessions (T2; Fig. 1) from both the rNMMS and contralateral sNMMS muscles. At the histological examination, all the ALS muscles displayed, regardless of the type of stimulation, a marked variability in fiber size because of numerous atrophic angulated fibers and sporadic groups of hypertrophic fibers (range of cross sectional fiber diameter: 8–86 μm in sNMMS muscle; 8–108 in rNMMS muscle) (Fig. 4a,b). Atrophy involved both slow and fast fiber types and was associated with fiber type grouping, which is consistent with the occurrence of previous reinnervation phenomena (Fig. 4c).

The morphometric analysis performed on sections immunostained with antibodies for either slow or fast myosin revealed that rNMMS significantly counteracted muscle atrophy in fast twitch fibers when compared with sNMMS (p < 0.001, regression model t-test). This effect was not observed in slow fibers, whose diameters instead displayed a slight, though significant, reduction in size (p < 0.01, regression model t-test) (Fig. 4d). These features were paralleled by a higher mean percentage of fast twitch fibers in rNMMS than in sNMMS muscles (66.10 ± 29.6% *vs* 49.22 ± 20.10%, p < 0.005, paired t-test).

### NMMS acts on the function of muscular nicotinic ACh receptors

**Figure 5:**
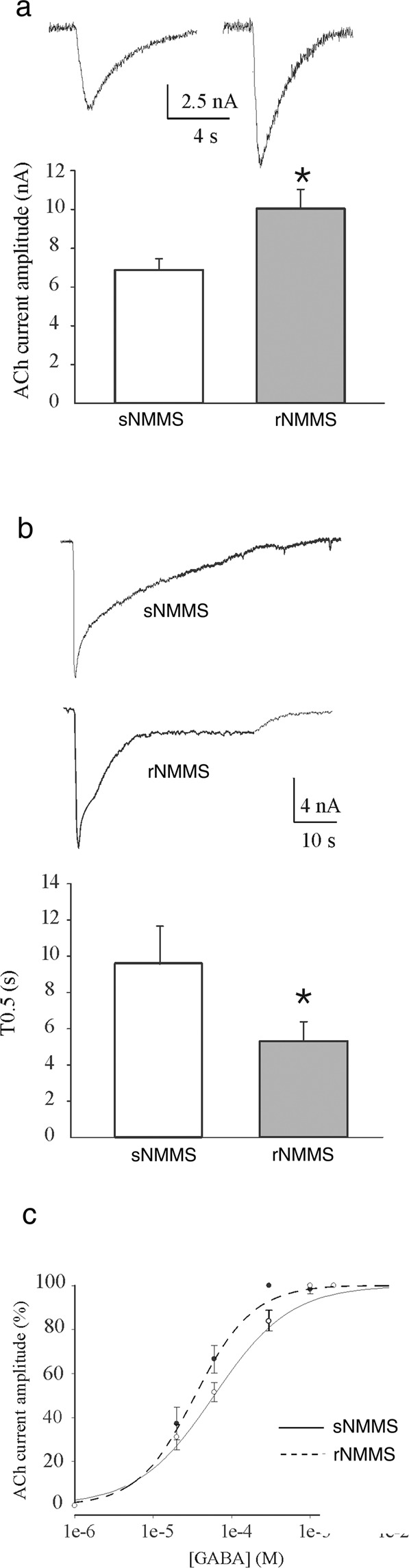
Electrophysiological analysis of ACh responses in sNMMS- and rNMMS. (**a**) The bar graphs show the mean ACh current amplitude (mean ± SEM) in sNMMS vs rNMMS injected oocytes. (Inset) Representative current traces from sNMMS (left) and rNMMS (right) injected oocytes. [ACh] = 500 μM; 4 s applications. *p < 0.05. Note the increase in current amplitude after rNMMS. (**b**) The bar graphs show the mean ACh current decay time (mean ± SEM) in sNMMS *vs* rNMMS injected oocytes. (Inset) Representative current traces from sNMMS (upper) and rNMMS (lower) injected oocytes. [ACh] = 500 μM; 30 s applications. *p < 0.05. Note the faster current decay time induced by rNMMS. (**c**) Averaged EC_50_ values in oocytes injected with membranes from sNMMS and rNMMS patients, showing an increase in ACh affinity after rNMMS treatment.

We used the membrane microtransplantation technique applied to Xenopus oocytes^14^ to overcome the technical and ethical difficulties to perform cellular electrophysiological recordings in muscles from ALS patients. The small amount of tissue collected from the muscle biopsy of 15 patients (about 10 mg of muscle tissue) proved to be sufficient to evoke ACh currents when membranes were transplanted in the oocytes (range: 1.6–67.4 nA; n = 283; 15 patients; unless stated otherwise, the ACh concentration was 500 μM and the holding potential −60 mV). We observed that the amplitude of the ACh-evoked currents (I_ACh_) was significantly higher in oocytes microtransplanted with rNMMS tissues than in untreated tissues (rNMMS: 10.61 ± 1 nA; n = 139; 15 patients vs sNMMS: 7.14 ± 0.6 nA; p = 0.036, Mann-Whitney rank sum test; n = 144; 15 patients) (Fig. 5a).

Moreover, we studied the decay time (T_0.5_) of the I_ACh_ during ACh long application in both sNMMS and rNMMS tissues. Our experiments showed that T_0.5_ (i.e. the time required for the I_ACh_ to reach half of its peak amplitude during the decaying phase) is faster following rNMMS than in sNMMS (rNMMS: 5.3 ± 1 s; n = 18; sNMMS: 9.6 ± 2 s; n = 16; p = 0.034, Mann-Whitney rank sum test; 4 out of 15 patients) (Fig. 5b). Taken together, these results indicate that rNMMS improves ACh function by modifying the AChR response. When we assessed ACh affinity in this study, we observed that the ACh dose-response curve for the rNMMS samples shifted to the left if compared with the sNMMS samples (sNMMS: 51 ± 0.4 µM; n = 18; vs rNMMS: 33.2 ± 2.7 µM; n = 16; p = 0.028, Mann-Whitney rank sum test; 4 out of 15 patients) (Fig. 5c).

### Molecular mechanisms activated in response to rNMMS treatment counteract muscle atrophy

To determine the adaptation changes in gene expression due to rNMMS, we performed a real time PCR (RT-PCR) analysis to quantify shifts in mRNA levels of a selected panel of genes involved in muscle growth and plasticity in both rNMMS and sNMMS samples of 7 patients. The keys factors involved in skeletal muscle adaptations and growth are insulin-like growth factor-1 (IGF-1)^15,16^ and myostatin^17,18^. IGF-1 and myostatin play opposing roles in regulating the size of skeletal muscle, with the former stimulating and the latter inhibiting muscle growth.

**Figure 6:**
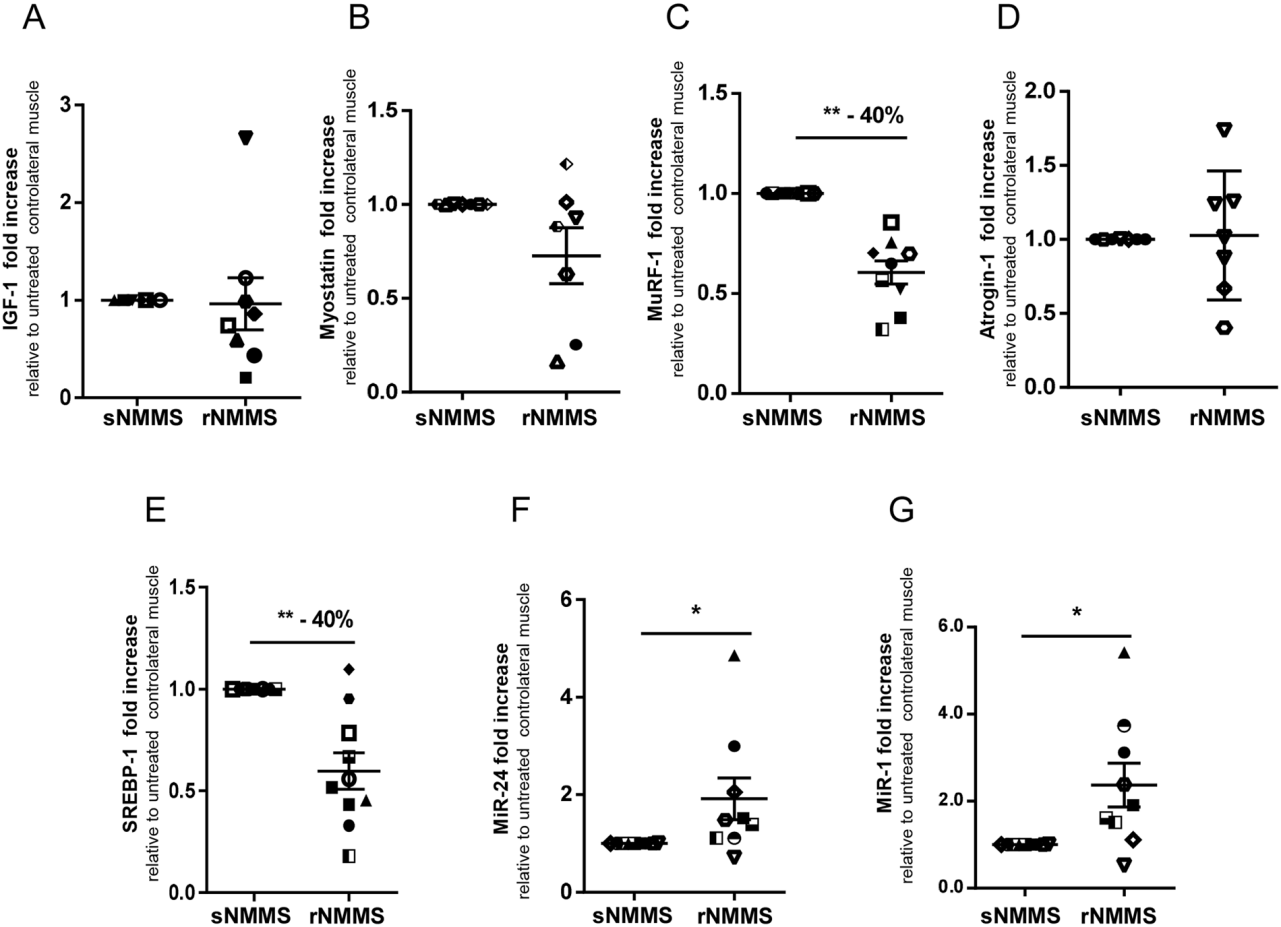
NMMS treatment counteracts muscle atrophy and enhances robustness and resistance to fibrosis in ALS muscles. (**A**) Real-time PCR analysis of IGF-1EA, (**B**) myostatin, (**C**) MuRF-1, (**D**) atrogin-1, (**E**) SREBP-1, (**F**) MiR-24, (**G**) MiR-1 transcript in rNMMS- and sNMMS-treated samples (**p < 0.01, *p < 0.05. The number above the graph indicates the percentage decrease in transcript expression in treated vs control samples. Data are represented as mean ± SEM).

Figure 6 shows that rNMMS did not induce any significant difference in the expression levels of either IGF-1 (Fig. 6A) or myostatin (Fig. 6B) (IGF-1 rNMMS: 0.96 ± 0.27; p = 0.38; myostatin rNMMS: 0.73 ± 0.15; p = 0.16, Wilcoxon matched-pairs rank test), thus suggesting that the trophic action of rNMMS acts on alternative pathways.

Transcriptional up- or down-regulation of atrophy-related genes is a characteristic feature of muscle atrophy^19^. The most critical players involved in muscle atrophy are MAFbx/atrogin-1 and MuRF-1, which are muscle-specific atrophy-related ubiquitin ligases responsible for increased protein degradation through the ubiquitin-proteasome system. We found a significant down-regulation of MuRF-1 and a reduced trend in atrogin-1 expression in the rNMMS samples post-treatment (Fig. 6C and D) (MuRF-1 rNMMS: 0.62 ± 0.05; p = 0.002; atrogin-1 rNMMS: 1.03 ± 0.16; p = 0.94, Wilcoxon matched-pairs rank test), which suggests that rNMMS preserved muscle mass by modulating protein catabolism. The maintenance of muscle mass is the result of the delicate balance between anabolic and catabolic processes. We thus investigated the level of SREBP-1, a transcription factor whose accumulation has been associated with a decrease in the protein synthesis rate and with the negative modulation of muscle protein content^20^. As shown in Fig. 6E, we observed a significant down-modulation in SREBP-1 in the treated muscle biopsies compared with untreated biopsies, (SREBP-1 rNMMS: 0.61 ± 0.08; p = 0.003; Wilcoxon matched-pairs rank test), thereby confirming that rNMMS counteracts muscle atrophy by down-modulating proteolysis and attenuating the expression of protein synthesis inhibitors.

To lend further support to this hypothesis, we evaluated other key players involved in the homeostatic maintenance of skeletal muscle, namely MiR-24 and MiR-1, which are strongly induced during myogenesis and are maintained at high levels in terminally differentiated muscle tissues^21^. Figure 6F and G show a statistically significant accumulation of both MiRNAs in treated muscle compared with untreated muscle (MiR-24 rNMMS: 1.91 ± 0.43; p = 0.019, MiR-1 rNMMS: 2.37 ± 0.50; p = 0.012, Wilcoxon matched-pairs rank test).

### NMMS treatment attenuates muscle denervation in ALS patients

**Figure 7:**
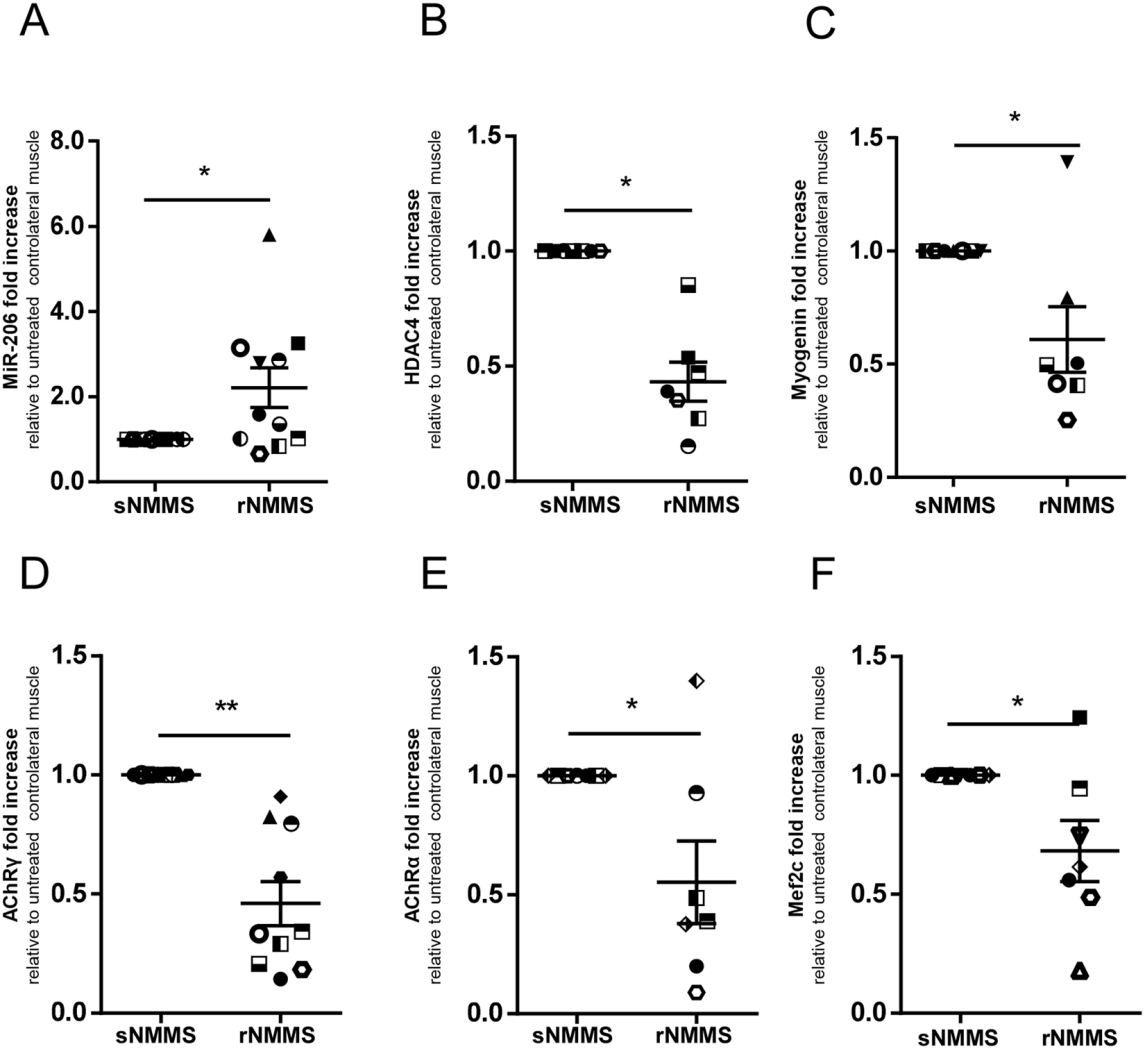
NMMS treatment stabilizes the neuromuscular end plate and preserves muscle fiber composition during disease progression. (**A**) Real-time PCR analysis of MiR-206, (**B**) HDAC4, (**C**) Myogenin, (**D**) AChRγ, (**E**) AChRα, (**F**) Mef2c transcripts in rNMMS- and sNMMS-treated samples (*p < 0.05 **p < 0.01. Data represent mean ± SEM).

Samples obtained from biopsies of 7 ALS patients display signs of denervation and reinnervation of muscle fibers and the upregulation of the γ subunit of AChR in ALS muscles^10^. Here we show that rNMMS modulated the regulatory circuit of muscle-nerve interplay^22^, up-regulating MiR-206 (Fig. 7A) and down-regulating HDAC4 (Fig. 7B), myogenin (Fig. 7C) as well as the γ (Fig. 7D) and α (Fig. 7E) subunits of the AChR (MiR-206 rNMMS: 2.21 ± 0.47; p = 0.019, HDAC4 rNMMS: 0.43 ± 0.08; p = 0.02; myogenin rNMMS: 0.61 ± 0.14; p = 0.047 AChR-γ rNMMS: 0.47 ± 0.10; p = 0.004 Wilcoxon matched-pairs rank test; AChR- α rNMMS: 0.55 ± 0.17; p = 0.02 t-test).

Mef2c is reported to be up-regulated in ALS muscle^23^ and to be reduced by MiR-206 upregulation in type II reinnervated fibers^24^. To verify whether MiR-206 up-regulation correlates with the modulation of Mef2c and with maintenance of muscle fiber composition, we investigated the transcript level of Mef2c in rNMMS-treated ALS samples and in sNMMS samples. Figure 7F shows a significant down-modulation in Mef2c transcript levels in the rNMMS arm compared with the sNMMS arm (Mef2c rNMMS: 0.68 ± 0.13; p = 0.04 t-test).

## Discussion

This study shows the efficacy of localized rNMMS treatment in improving muscle strength, while reducing muscle atrophy and alteration in molecular markers of the functional interplay between muscle and nerve. A significant improvement in muscle strength was observed in the treated arm as tested by the MRC Muscle Scale and by dynamometer. The increase in muscle strength measured by dynamometer was significantly higher in the treated compared to untreated muscle at T2. These results suggest that rNMMS induces a focal improvement in muscle strength. This effect progressively decreased after the end of rNMMS (T3) for the dynamometer and the MRC Muscle Scale, probably because of the natural progression of the disease. Interestingly, the CMAP amplitude remained the same before and after stimulation, thereby suggesting that the improvement was not related to reinnervation phenomena^25^. Moreover, the increase in muscle strength was limited to the treated muscle, which indicates that the effect of rNMMS is localized, with no action being exerted on the distal muscles owing to the decay in the magnetic field.

Muscle atrophy and weakness are among the first signs of ALS disease, even before motor neuron degeneration is observed^26^. A modulation of muscle protein catabolism as down-modulating of MuRF-1 and atrogin-1 expression, an attenuation of the expression of protein synthesis inhibitors as SREBP-1, and an increase of factors involved in the homeostatic maintenance of skeletal muscle, as MiRNAs were evidenced at the end of the NMMS treatment.

One important characteristic of skeletal muscles is their plasticity, which is needed to modulate their properties in response to a wide range of external factors, including physical and motor-neuron activity, changes in hormone levels and oxygen and nutrient supply. One of the pathological features of ALS is a change in the muscle fiber profile, with a selective loss of fast-twitch fibers^27–29^ that is likely to be due to the selective vulnerability of fast fatigable and fast fiber-innervating motor neurons^30^. The morphometric analysis evidenced a higher mean percentage of fast twitch fibers in treated than in untreated muscles, thereby suggesting that rNMMS preserves fast fibers and consequently attenuates muscle atrophy.

Previous studies demonstrated that the diaphragm pacing had a deleterious effect on ALS patients with respiratory failure, as measured by overall survival; a possible explanation of this effect is that pacing may cause excessive muscle fatigue or that asynchrony between pacing-induced diaphragmatic contraction and patient- and/or NIV-triggered breaths may represent an issue. On the other hand, in line with our results, diaphragm pacing may induce a shift from a predominance of efficient type I fibres to inefficient type IIb fibres^31^. In contrast to the electrical stimulation, no significant muscle twitch was observed during the rNMMS sessions; this data – altogether with the lack of changes observed in the CMAP amplitudes registered before and after the stimulation protocol – suggests that the effect of the magnetic field is not likely exerted on motor nerves, but directly on muscle fibers or on nerve-muscle connection.

Tirasemtiv is a fast skeletal muscle troponin activator (FSTA), which increases the contractile sarcomeric response by selectively binding to the fast skeletal muscle troponin complex, thus slowing the rate of calcium ion release and sensitizing the sarcomere to calcium^32^. Despite Tirasemtiv improved physical function in SOD1 transgenic mouse models of ALS, in a phase IIb trial the primary endpoint showed no treatment effect. Nevertheless, the primary endpoint and most of the secondary endpoints concerned certain respiratory milestones of disease progression, instead of direct muscle function measures. It is important to underline that quantitative muscle strength declined significantly more slowly on Tirasemtiv group.

Since rNMMS counteracts morphological changes and reduces atrophy in ALS muscles, we wondered whether rNMMS also modifies the electrophysiological function of nicotinic AChRs, which play a key role in muscular contraction. Our results confirm that rNMMS promotes the maintenance of AChR, increasing ACh affinity and restoring nAChR function to levels resembling those observed in non-ALS denervated patients. Indeed, we observed an improvement in the efficacy of ACh, as shown by the increase in ACh current amplitude after rNMMS treatment. In addition, faster current decay correlates with the change in ACh affinity, which suggests that AChRs may be more inducible at low neurotransmitter doses. The improved electrophysiological function of the AChR might be a result of the down-modulation of proteolysis and of fibrotic processes induced by rNMMS and might partially explain the beneficial effects exerted by rNMMS stimulation on the contractile capability of the diseased muscle. The maintenance of the functional connection between nerve and muscle was revealed by the modulation of γ subunit containing AChR (AChRγ) after rNMMS treatment. AChRγ is expressed at high levels in muscle during embryonic development and perinatally, whereas its expression is low or undetectable in normal active or disused adult muscle. By contrast, AChRγ expression increases in denervated muscle or under conditions that alter NMJ functionality^6,33^. Our data demonstrate that rNMMS treatment stabilizes the neuromuscular end plate and muscle maintenance. The mini-regulatory circuit that controls the muscle-nerve interplay involves the class IIa histone deacetylase-4 (HDAC4) and myogenin, a bHLH transcription factor whose expression is down regulated in adult skeletal muscle and induced in response to denervation^22,34,35^, which in turn promotes the activation of MuRF-1 and atrogin-1^36^. Upon reinnervation signals, myogenin activates a key regulator of the bidirectional signaling between motor neurons and skeletal muscle fibers, namely MiR-206. MiR-206 is reported to be dramatically induced in the mouse model of ALS and in denervated muscle fibers, in which it establishes a negative feedback loop that promotes muscle reinnervation and repression of HDAC4 expression^37^. We demonstrated that rNMMS up-regulates MiR-206, which in turn modulates HDAC4, myogenin and AChRγ, all of which act as important regulators of the signaling that detects nerve activity within the muscle. Our data clearly indicate that rNMMS improves muscle function and mass by regulating key factors and the signaling involved in nerve-muscle connection. Overall, this study represents and provides a first evidence that local rNMMS treatment of ALS patients enhances muscle strength and may consequently improve the daily life of such patients.

Interestingly, the clinical benefits of rNMMS result in a better hand function of the stimulated arm even after stimulation ends. It is therefore conceivable that this technique may be further developed to improve fine motor skills activities by increasing both the duration and the modality of NMMS stimulation. The recovery of some activities of daily living, such as holding a pencil or a spoon, are also considered to be critical issues by ALS treated patients owing to the psychological consequences associated with the inability to perform such activities.

This is in keeping with the concept of health-related quality of life (HRQoL) proposed by Shumaker and Naughtonas: “people’s subjective evaluations of the influences of their current health status, health care, and health-promoting activities on their ability to achieve and maintain a level of overall functioning that allows them to pursue valued life goals, and that is reflected in their general well-being”^38^. Thus, any therapeutic approach aimed at preserving HRQoL by improving some aspect of independence may be considered to be “clinically meaningful”.

The clinical data were supported by physiological and molecular changes that demonstrated the activation of local circuits that attenuate muscle weakness. Our data further support the so-called “dying-back” phenomenon and highlight the potential of therapeutic interventions designed to attenuate muscle dysfunction, NMJ changes and possibly even disease progression in a sort of “saving-back” process.

**Figure 8:**
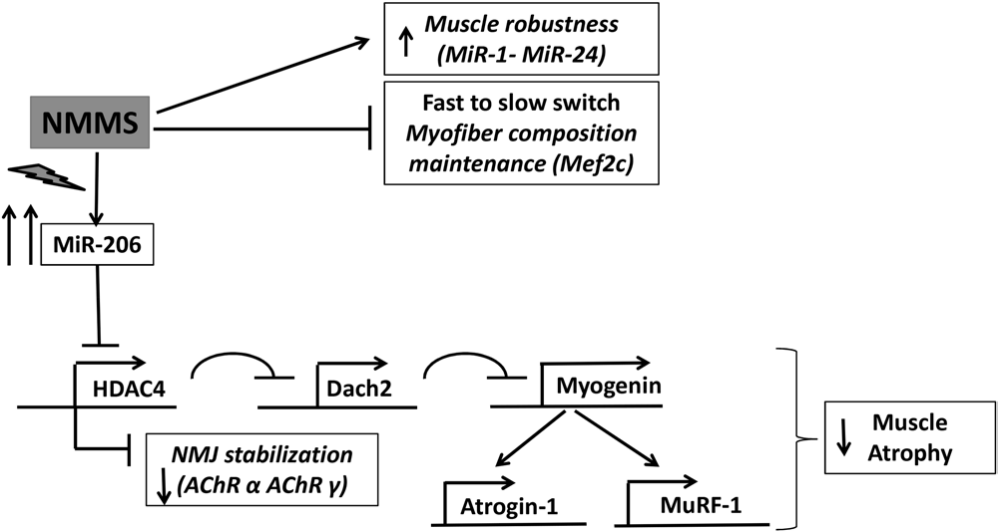
Schematic representation of NMMS effect on ALS muscle decline.

Thus, our work is consistent with a model (Fig. 8) in which rNMMS treatment prevents muscle atrophy, preserves fiber type composition and stabilizes the neuromuscular junction, thereby enhancing the robustness of the muscles in ALS patients.

Undoubtedly, our study has important limitations. The small number of bioptic samples did not allow some statistical analyses or stratification for all variables, and an excessive multiple statistical analyses were also used seen the several clinical, molecular and morphological patterns investigated. We have chosen the percutaneous needle biopsy technique to make the patient’s collection less unpleasant, but the material obtained was not sufficient to carry out completely the histological, histomorphometric and RNA analysis studies. Moreover, in spite of these aspects, the results obtained with different experimental approaches all lead to the same conclusion about the objective support of clinical improvement of muscle strength, through counteracting muscle atrophy (increase of growth factors that support muscle trophism), down-modulating of proteolysis (reduction of factors that favor atrophy) and increasing the efficacy of nicotinic AChRs. These unidirectional results reassure us on the validity of the results obtained.

Although optimism about the use of rNMMS as a novel translational approach to the treatment of ALS, this work is a proof of concept study without an immediate clinical translation. Moreover, this intervention should be analyzed on more global ALS progression parameters (overall survival, ALSFRS-R score, pulmonary function tests). It is possible that the same technique might be difficult to adapt to different clinical forms of ALS, given the underlying heterogeneity of the disease (i.e. bulbar-onset patients, upper motor neuron predominant involvement, fast progression of the disease). Further studies, on both ALS patients and animal models, are needed to shed light on how local muscle-NMJ treatment can activate survival pathways at the spinal and cortical levels.

## Methods

This single-center randomized double blind controlled study was designed to evaluate the efficacy of NMMS in spinal-onset (ALS) patients. Participants were subsequently randomly assigned in 1:1 ratio. Clinical trial was registered with number: NCT03618966; date of registration: August 07, 2018.

### Patients

Subjects were recruited from the ALS Center of Policlinico Umberto I, Sapienza University of Rome from November 2014 to November 2017. Inclusion criteria were: (i) diagnosis of probable or definite spinal-onset ALS according to electrodiagnostic criteria^39^, (ii) moderate degree of muscle atrophy, and (iii) bilateral symmetric muscular deficit in flexor carpi radialis muscle or flexor digitorum profundus muscle, as defined by a MRC Muscle Scale score of 3-4/5. Exclusion criteria included history of epilepsy or severe headaches, pregnancy or breast-feeding, the presence of an implanted cardiac pacemaker, neurostimulators, surgical clips or medical pumps, or any other comorbid condition that might prevent the patient from completing the study. Moreover, the patients previously treated with Transcranial Magnetic Stimulation (TMS) or NMMS were also excluded. The patients’ clinical characteristics are summarized in Table 1. The study was approved in 2014 by the Institutional Review Board of Policlinico Umberto I (reference 3314/25.09.14; protocol no. 1186/14) and performed in accordance with the Declaration of Helsinki. All the patients provided written informed consent prior to inclusion in the study.

**Table 1:** Clinical characteristic at T0 of the 22 patients included in the study (19 males. 3 females). Data are expressed as mean ± standard deviation (SD).

	MEAN	SD
Age (years)	61.27	13.44
Disease duration (months)	26.82	15.43
ALSFRS-R (tot)	35.55	6.82
FVC (%)	80.14	16.91
**MRC Muscle Scale (upper limbs)**		
stimulated	31.73	4.17
not stimulated	29.59	5.25
**MRC flexor carpi radialis**		
stimulated	3.95	0.57
not stimulated	3.63	0.85
**Dynamometer (Kg)**		
stimulated	22.0	6.2
not stimulated	19.9	5.8
**CMAP amplitude (mV)**		
APB muscle		
stimulated	3.8	2.93
not stimulated	3.24	3.3
**Flexor carpi radialis muscle**		
stimulated	11.48	5.17
not stimulated	9.59	5.81

### Experimental design

The follow-up for each patient lasted for 4 weeks. At the baseline visit, patients were randomized in two groups on the basis of a sequential number from a computer-generated random list. One group received real stimulation (rNMMS) in the right arm and sham stimulation (sNMMS) in the left arm in the same daily session; the other group received rNMMS in the left arm and sNMMS in the right arm. All the patients underwent a medial nerve conduction study (NCS) at T0 and T2 and a clinical examination at time T0, T1, T2 and T3 (T0 was the first recording of clinical strength and neurophysiological parameters before stimulation, T1 after one week of stimulation, T2 after two weeks of stimulation, T3 after thirty days of stimulation) (Fig. 1). The clinical examination included i) a handgrip strength test to measure the maximal isometric strength of the hand and flexor forearm muscles, and ii) MRC Muscle Scale to manually test the upper limb muscles. A needle muscle biopsy was performed bilaterally, under a local anesthetic, to obtain a flexor carpi radialis muscle sample for the histological, physiological and molecular studies at T2. After three months from the end to the study, the patients underwent a new clinical visit to also evaluate potential side effects. Patients and physicians who performed all examinations were blinded to the allocation of real/sham intervention, except one physician (MI), who did the randomization process and performed the NMMS treatment supported by a neurophysiopathologist technician (GT). The subjects involved in the study, namely people who performed the clinical evaluation (CC, EO, MCG), the electrophysiological examinations (MC, VF), the histological and histomorphometric analysis (AP, BC, CG), the RNA preparation and real-time analysis (AM, GD, EL) and the electrophysiological recordings (GR, PC, EP, CR) were blinded to the subject’s treatment.

Motor NCS was performed using a Micromed Myoquick 1400 EMG machine (Micromed S.p.A., Treviso, Italy); Ag/AgCl surface electrodes were applied according to a conventional belly-to-tendon method. Filter settings were 5 Hz–5 kHz, sweep duration was 30 ms and sensitivity was 1 mV/division. The median nerve was stimulated at the elbow, bilaterally, and the maximal cMAP amplitude was recorded from the APB and flexor carpi radialis muscles. To ensure that the electrodes were positioned correctly at T0 and T2, a henna pinpoint tattoo was applied to mark the center of the muscle being studied. The MRC Muscle Scale grades the patient’s strength in 8 pairs of muscles for the upper limbs on a scale of 0–5. The handgrip strength test was performed by asking the patient to hold and then squeeze, with the arm at a right angle and the elbow by the side of the body, a dynamometer with maximal effort for three seconds; the mean value from three trials was recorded in Kg for each side, with 30-second recovery period after each trial.

No changes to methods after trial commencement were needed.

### Primary and Secondary Outcomes

The primary outcome was the evaluation of the efficacy and safety of NMMS in improving the muscle strength of patients affected by ALS according to MRC-score. Secondary outcomes included the effect of rNMMS on: 1. the muscular strength measured with handgrip dynamometer; 2. the electrophysiological changes (CMAP) to define the physiological mechanisms of the rNMMS; 3. the analysis of ACh evoked currents from bioptic muscles; 4. the histomorphometric parameters of muscle of ALS patients, analyzing muscle fiber type composition, muscle atrophy and wasting; 5. the changes in gene expression of a selected panel of genes involved in muscle growth and plasticity through a real time PCR (RT-PCR) analysis. Outcome measures were completed at baseline and at the end of each treatment phase. Potential adverse events, experienced by the patients, were monitored during the period of treatment and during the 3 months follow-up period.

### NMMS

All the patients received daily real and sham repetitive NMMS sessions, for five days a week for two consecutive weeks. Real NMMS was delivered by means of a high-frequency magnetic stimulator (Magstim Rapid – The Magstim Company Ltd., Whitland, South West Wales, UK) connected to a conventional cooled coil (Magstim AFC Coil). The magnetic stimulator was placed over the flexor muscles of the forearm. rNMMS was delivered at a 5-Hz frequency at 100% of the maximum intensity in 140 trains of 50 stimuli; the inter-train interval was 15 seconds. Sham NMMS was performed using a sham coil inserted in the same magnetic stimulator (Magstim AFC Coil), inducing a very shallow magnetic field. This weak magnetic field replicates the sensation of rNMMS without deep activation and with the production of similar acoustic sensations and with mechanical skin perceptions as rNMMS^40^.

### Bioptical procedure

A needle muscle biopsy (TSK Acecut Biopsy Needle 14Gx150 mm) under a local anesthetic was performed bilaterally from flexor carpi radialis muscle for histological, physiological and molecular studies in the treated and in the untreated arms at the end of the NMMS treatment.

The biopsy site was infiltrated with about 2 cc of lidocaine intradermally and subcutaneously with a 25 G needle to anesthetize the subcutaneous tissue and muscle fascia in order to avoid distorting the muscle sample to be biopsied. The needle was introduced perpendicularly into the muscle, and the inner cannula was open for about 30–45 seconds, allowing the suction to draw in a muscle sample. The procedure was repeated 2–3 times until an adequate sample was obtained.

### Histological analysis

Serial 5-micrometre (µm) cryostat sections from each biopsy were stained with hematoxylin and eosin (HE) for the morphological analysis and immunostained with antibodies again fast, slow and fetal myosin (Novocastra, MI, Italy) to evaluate the distribution of fiber types. A total of 14 samples were analyzed (rNMMS and sNMMS arms from 7 patients).

### Histomorphometric analysis

To quantify the degree of muscle atrophy, a histomorphometric analysis was performed on 4 out of 7 selected patients and rNMMS and sNMMS muscles immunostained with fast or slow myosin. The muscle was selected only if afforded the possibility to count at least 50 fibers (up to 140) per muscle section. The frequency distribution of the slow- and fast-twitch fiber diameter was evaluated for each muscle biopsy. High-resolution images were acquired at 10 and 20X magnification using a digital camera (Olympus) and analyzed by means of a dedicated software (ImageJ 1.47 v, Wayne Rasband National Institutes of Health, USA). The effect of rNMMS treatment on the diameter size was evaluated for each fiber type by means of a regression model that accounted for patient heterogeneity.

### RNA preparation and real-time analysis

Total RNA from human muscle biopsies of 7 patients was isolated by TRIzolTM reagent (Thermo Fisher Scientific); total RNA (1 μg) was reverse-transcribed using the QuantiTect Reverse Transcription Kit (Quiagen), while 10 ng of RNA were reverse-transcribed using the TaqMan micro RNA Reverse Transcription Kit (Thermo Fisher Scientific). Quantitative PCR was performed using the ABI PRISM 7500 SDS (Thermo Fisher Scientific), Taqman universal MMIX II (Thermo Fisher Scientific) and TaqMan probe (Thermo Fisher Scientific). The quantitative RT-PCR sample value was normalized for the expression of GAPDH and U6 snRNA for mRNA and microRNA, respectively. The relative expression was calculated using the 2−ΔΔCt method^41^ and reported as a fold change.

### Membrane reparation, injection procedures, and electrophysiological recordings from oocytes

Human muscle samples (about 10 mg) of 15 patients were frozen in liquid nitrogen immediately after biopsy and stored at −80 °C. Membranes were prepared as previously detailed^14^. Membrane currents were recorded from voltage-clamped oocytes 24–48 h after injection using two microelectrodes filled with 3 M KCl. The oocytes were placed in a recording chamber (volume, 0.1 mL) and perfused continuously, 9–10 mL/min, with oocyte Ringer’s solution (OR) at room temperature (20–22 °C). Unless otherwise specified, the oocytes were voltage clamped at −60 mV and acetylcholine (ACh) was 500 μM, 4 s application. In a subset of experiments (4 out of 15 patients, randomly selected) we applied ACh for 30 s to measure the ACh current desensitization defined as the time taken for the current to decay from its peak value to half-peak value (T0.5). Acetylcholine (ACh; Tocris) was dissolved in OR just before use. Data were analyzed using Sigma Plot 12 software and are given as means ± SEM; datasets were considered statistically different when p < 0.05. All the electrophysiological experiments were performed in blind fashion.

## Statistical Methods

Based on a previous study on measurement of muscle strength in healthy seniors before and after electrical stimulation training, also using the variation of muscle function after treatment with NMMS as a primary outcome, and assuming a pooled standard deviation of 4.9 units, the sample size was defined 11 patients per group to obtain a statistically significant result with 80% of power and a level of significance of 5% (two sided)^11^. Taking into account a 40% drop-out, it was decided to enroll at least 16 patients per group.

All details related to statistical tests, statistical parameters, including sample sizes and significance are reported in the results section. Graph values are reported as mean ± standard error of the mean (SEM). Statistical evaluation for the study of muscular nicotinic AChRs was performed using Mann-Whitney U test; for morphometric analysis of 4 selected biopsies was performed using the regression model t-test; for mRNA levels of selected panel of genes was performed by Wilcoxon matched-pairs rank test; for the muscle strength was performed using a RM ANOVA with a Greenhouse-Geisser correction (sphericity not assumed). Data were considered statistically significant when p < 0.05. Statistical analysis was performed using either Sigmaplot 12 or GraphPad PRISM 6 software.

## Data Availability

All the data and protocol details are available from the corresponding authors upon request.
